# Clinical‐Grade Isolated Human Kidney Perivascular Stromal Cells as an Organotypic Cell Source for Kidney Regenerative Medicine

**DOI:** 10.5966/sctm.2016-0053

**Published:** 2016-09-20

**Authors:** Daniëlle G. Leuning, Marlies E.J. Reinders, Joan Li, Anna J. Peired, Ellen Lievers, Hetty C. de Boer, Willem E. Fibbe, Paola Romagnani, Cees van Kooten, Melissa H. Little, Marten A. Engelse, Ton J. Rabelink

**Affiliations:** ^1^ Department of Nephrology, Leiden University Medical Centre, Leiden, The Netherlands; ^2^ Einthoven Laboratory of Vascular Medicine, Leiden University Medical Centre, Leiden, The Netherlands; ^3^ Institute for Molecular Bioscience, University of Queensland, Brisbane, Queensland, Australia; ^4^ Excellence Centre for Research, Transfer and High Education for the Development of DE NOVO Therapies, University of Florence, Florence, Italy; ^5^ Department of Biomedical, Experimental, and Clinical Sciences, University of Florence, Florence, Italy; ^6^ Department of Immunology and Hematology, Leiden University Medical Centre, Leiden, The Netherlands; ^7^ Murdoch Childrens Research Institute, Parkville, Melbourne, Victoria, Australia; ^8^ Department of Paediatrics, University of Melbourne, Melbourne, Victoria, Australia

**Keywords:** Tissue‐specific stem cells, Tissue regeneration, Stromal cells, Pericytes, Mesenchymal stromal cells, Kidney, Clinical translation, Cellular therapy

## Abstract

Mesenchymal stromal cells (MSCs) are immunomodulatory and tissue homeostatic cells that have shown beneficial effects in kidney diseases and transplantation. Perivascular stromal cells (PSCs) identified within several different organs share characteristics of bone marrow‐derived MSCs (BM‐MSCs). These PSCs may also possess tissue‐specific properties and play a role in local tissue homeostasis. We hypothesized that human kidney‐derived PSCs (hkPSCs) would elicit improved kidney repair in comparison with BM‐MSCs. Here we introduce a novel, clinical‐grade isolation method of hkPSCs from cadaveric kidneys by enriching for the perivascular marker, NG2. hkPSCs show strong transcriptional similarities to BM‐MSCs but also show organotypic expression signatures, including the HoxD10 and HoxD11 nephrogenic transcription factors. Comparable to BM‐MSCs, hkPSCs showed immunosuppressive potential and, when cocultured with endothelial cells, vascular plexus formation was supported, which was specifically in the hkPSCs accompanied by an increased NG2 expression. hkPSCs did not undergo myofibroblast transformation after exposure to transforming growth factor‐β, further corroborating their potential regulatory role in tissue homeostasis. This was further supported by the observation that hkPSCs induced accelerated repair in a tubular epithelial wound scratch assay, which was mediated through hepatocyte growth factor release. In vivo, in a neonatal kidney injection model, hkPSCs reintegrated and survived in the interstitial compartment, whereas BM‐MSCs did not show this potential. Moreover, hkPSCs gave protection against the development of acute kidney injury in vivo in a model of rhabdomyolysis‐mediated nephrotoxicity. Overall, this suggests a superior therapeutic potential for the use of hkPSCs and their secretome in the treatment of kidney diseases. Stem Cells Translational Medicine *2017;6:405–418*

**Significance Statement**

The present study provides a novel clinical‐grade isolation method of human kidney‐derived perivascular stromal cells (hkPSCs). The hypothesis was that, due to tissue‐specific imprinting, hkPSCs are more potent in renal repair compared to human bone marrow‐derived mesenchymal stromal cells. The present study shows that hkPSCs indeed show distinct functional properties with respect to epithelial wound healing, interstitial integration, and amelioration of kidney injury. Therefore, these results suggest significant potential for hkPSCs as therapeutic candidates for the treatment of kidney diseases.

## Introduction

Mesenchymal stromal cells (MSCs) are immune modulatory and antifibrotic cells originally isolated from the bone marrow (BM) and are characterized by their spindle‐shaped morphology and ability to adhere to plastic. BM‐MSCs are able to differentiate into fat, bone, and cartilage and express the stromal markers CD73, CD90, and CD105 while being negative for CD34 and CD45 1, 2. In several experimental models of kidney disease (among others, cisplatin, glycerol, and ischemia‐induced injury), MSC treatment enhanced tissue repair and reduced fibrosis 3, 4. In a transplantation model, MSC therapy could prolong graft survival, and a regulatory T‐cell‐dependent tolerance was observed 5. These promising results led to the first clinical trials with BM‐MSCs in renal transplantation. Although the group sizes were small and the studies were mainly set up to show safety and feasibility of MSC therapy, the first results suggest an immunomodulatory effect of BM‐MSC therapy 6, 7, 8, 9.

Previously, it has been shown that perivascular stromal cells positive for NG2, CD146, and PDGF‐R‐β with characteristics similar to BM‐MSCs exist within many different solid organs, including skeletal muscle, pancreas, adipose tissue, and placenta 10. Because of the perivascular location of these cells, close interaction is possible with several cell types, including endothelial cells, epithelial cells, resident macrophages, dendritic cells, and recruited inflammatory cells. Therefore, these cells are most likely important for tissue homeostasis and control of repair processes and inflammation 11.

MSC‐like cells could also be isolated from murine kidneys on the basis of Sca‐1 expression. These renal Sca1^+^Lin^−^CD45^−^ cells showed a unique phenotype and immunomodulatory potential 12, 13, 14. Murine kidney colony‐forming cells (kCFU‐F) were also Sca‐1^+^, and although these kCFU‐F have a comparable stromal marker expression and trilineage differentiation potential in comparison with BM‐MSCs, there is a distinct gene and protein expression profile 15. This suggests that although kCFU‐F and BM‐MSCs may look similar, functionally there may be differences. Li et al. showed that these cells are indeed different from BM‐MSCs 16. In particular, the cell fraction isolated on the basis of expression of HoxB7, a collecting duct marker, was able to undergo epithelial‐to‐mesenchymal transition, and upon delivery into neonatal mice kidneys, these cells integrated back into the collecting duct, whereas BM‐MSCs lacked this capacity 16.

We hypothesized that perivascular stromal cells can also be isolated from the human kidney (hkPSCs) in a clinical‐grade manner and that these cells have tissue‐specific functions. Extensive characterization of hkPSCs, both in vitro and in vivo, show a capacity for these cells to stabilize endothelial cells, reduce renal injury, and support tubular repair, the latter to a greater extent than BM‐MSCs, suggesting indeed organotypic properties.

## Materials and Methods

### Isolation and Expansion of Clinical‐Grade Human Kidney‐Derived Perivascular Stromal Cells

Cells were isolated from human transplant‐grade kidneys discarded for surgical reasons by using clinical‐grade protocols, enzymes, and products. Research consent was given for all kidneys, and the study was approved by the local medical ethical committee and the ethical advisory board of the consortium.

Kidneys were flushed with University of Wisconsin (UW) cold storage solution (Bridge to Life, Elkhorn, WI, http://www.bridgetolife.com) containing heparin (Leo Pharma, Ballerup, Denmark, http://www.leo‐pharma.us) directly after surgery and stored on ice. Within 30 hours, kidneys were flushed again with UW, and the perirenal fat and kidney capsule were removed. The renal artery was cannulated, and the kidney was perfused via a pump‐driven (Masterflex Applikon, Schiedam, The Netherlands, http://www.masterflex.nl) recirculation system at 37° with Dulbecco's modified Eagle medium (DMEM)‐F12 (Lonza, Basel, Switzerland, http://www.lonza.com) at 300 ml per minute. Afterward the kidney was perfused with collagenase (2,500 units, NB1; Serva, Heidelberg, Germany, http://www.serva.de) and DNAse (2.5 ml Pulmozyme; Genentech, San Francisco, CA, http://www.gene.com) at 37°C with a flow of 300 ml per minute. After approximately 30 minutes, the tissue was digested, and after gentle massage the resulting cell suspension was collected and washed in DMEM‐F12 containing 10% fetal calf serum. Cells were then either directly put into culture or frozen in liquid nitrogen. The complete standard operation procedure (SOP) for the isolation procedure can be found in the supplemental online data.

Kidney cell suspensions were cultured in alphaMEM (αMEM; Lonza) containing 5% platelet lysates, glutamine (Lonza), and penicillin/streptomyzine (Lonza), and cells were cultured in tissue culture flasks until confluence was reached. At passage 1, cells were trypsinized, and NG2 cell enrichment was performed using MACS, according to manufacturer's protocol (Miltenyi Biotech, Gladbach, Germany, http://www.miltenyibiotec.com). The NG2‐positive fraction was cultured in αMEM containing 5% platelet lysate. The cultures were maintained at 37°C and 5% carbon dioxide. Half the medium was refreshed twice a week. When the cells reached confluence, the cells were collected using trypsin (Lonza) and replated at 4 × 10^3^ cells per cm^2^. Experiments were performed with fluorescence‐activated cell sorting (FACS)‐confirmed homogeneous NG2‐positive hkPSCs between passages 4 and 8, cultured in αMEM 5% platelet lysate unless stated differently.

### Isolation and Expansion of Human Bone Marrow‐Derived Mesenchymal Stromal Cells

Ethical committee approval and written consent from the donors was obtained for the aspiration of human bone marrow. Heparinized bone marrow was aspirated under local or general anesthesia. The mononucleated cell fraction was isolated by Ficoll density gradient separation and plated in tissue culture flasks at a density of 160 × 10^3^ mononucleated cells per cm^2^ in αMEM (Lonza), supplemented with penicillin/streptomycin (Lonza) and 5% platelet lysate. The cultures were maintained at 37°C, 5% carbon dioxide. Half the medium was refreshed twice a week. When the MSC colonies or cultures reached confluence, the cells were collected using trypsin (Lonza) and replated at 4 × 10^3^ cells per cm^2^. Experiments were performed between passages 4 and 8.

### Morphology and Immunophenotype Analysis

The expanded cell populations were characterized by morphology (spindle‐shaped cells), which was imaged with an inverted bright‐field microscope (Leica DFC 295; Leica Biosystems, Wetzler, Germany, http://www2.leicabiosystems.com/). For immunophenotyping, the cells were stained for NG2, PDGF‐R‐β, CD146, CD73, CD90, CD105, CD31, CD34, CD45, CD56, human leukocyte antigen (HLA) class I (ABC), and HLA class II (DR). All specific fluorochrome‐labeled antibodies and isotype controls were purchased from BD Bioscience (Franklin Lakes, NJ, https://www.bdbiosciences.com), except for CD105 (Ancell Corporation, Stillwater, MN, http://www.ancell.com).

### Microarray Sample Preparation and Data Analysis

RNA was isolated from biological triplicates using Trizol reagent (Thermo Fisher Scientific Life Sciences, Waltham, MA, http://www.thermofisher.com) and the RNeasy kit (Qiagen, Hilden, Germany, https://www.qiagen.com), according to the manufacturer's protocol. The quality and quantity of RNA were assessed with a Nanodrop spectrophotometer (Thermo Fisher Scientific Life Sciences) and a Bioanalyzer (Agilent Technologies, Santa Clara, CA, http://www.agilent.com). Gene expression profiling was performed by Aros Applied Biotechnology (Aarhus, Denmark, http://arosab.com). cDNA and cRNA synthesis, labeling, and subsequent hybridization on the human HT12 V4 gene expression beadchips (Illumina Inc., San Diego, CA, http://www.illumina.com) were performed according to the manufacturer's protocols. The beadchips, targeting more than 47,000 gene transcripts, were scanned with the iSCAN system (Illumina Inc.), and fluorescence intensities were uploaded into GenomeStudio Software (Illumina Inc.). Genes with a detection *p* value of >.05 for all samples were excluded. Average signals of >200 in either the BM‐MSCs or hKPSCs were considered above background levels. Subsequent data were quantile normalized, and the Pearson's correlation coefficient was calculated (*r* ^2^). Differential expression was analyzed with the gene expression module of Genome Studio (Illumina). For the table of the top differential expressed genes, we sorted genes on the highest difference score. Differential scores were calculated from the differential *p* value in Illumina software with the following formula: DiffScore = 10 × sgn (μcond − μref) × log 10 *p*. False discovery rates were calculated according to Benjamini and Hochberg for a total number of 8,462 transcripts 17. All genes in the top five up‐ and downregulated genes showed significant differential expression. The delta average signal of BM‐MSCs versus hkPSCs was set to 200 (which is the threshold of the measurement), and 2,600 genes selected were analyzed for clustering in R software. Clustering in R software was also performed for 27 homeobox genes. Expression of HoxD10 and HoxD11 was confirmed by quantitative real‐time polymerase chain reaction (qPCR) in duplo of the same biological triplicates. qPCR was performed using iQ SYBR Green Supermix on iCycler real‐time detection system (Bio‐Rad, Hercules, CA, http://www.bio‐rad.com), as per the manufacturer's instructions. The real‐time PCR primers of HoxD10 are AGACAGTTGGACAGATCCGAA(fw) and CGAAATGAGTTTGTTGCGCTTAT (rv) and of HoxD11 are TCGACCAGTTCTACGAGGCA (fw) and AAAAACTCGCGTTCCAGTTCG (rev). The amplification reaction volume was 12.5 µl in total, consisting of 6.25 µl iQ SYBR Green PCR master mix, 0.5 µl primers, 2.5 µl cDNA, and 3.25 µl water. Messenger RNA (mRNA) level was normalized to the housekeeping gene glyceraldehyde‐3‐phosphate dehydrogenase.

### Trilineage Differentiation Potential

hkPSCs and BM‐MSCs were cultured in adipogenic, osteogenic, and chondrogenic medium according to the manufactures protocols (Lonza). After 3 weeks of culture, in the adipogenic differentiation assay, lipid droplets were stained using Oil Red O, and in the osteogenic differentiation assay, calcium depositions were stained with Alizarin Red. For chondrogenic differentiation, cell pellets were formalin fixed (4% paraformaldehyde [PFA] O/N) and embedded in paraffin. Subsequently, 5‐µm sections were deparaffinized, rehydrated, and stained with 1% toluidine blue for 20 minutes. All differentiation assays were analyzed with an inverted bright‐field microscope (Leica DFC 295; Leica Biosystems).

### Cytokine Excretion, Peripheral Blood Mononuclear Cell Isolation, and Proliferation Assay

hkPSCs and BM‐MSCs of 3 different donors were plated in flat‐bottom 96‐well plates, and after 5 days of culture, supernatants were harvested, and cytokine expression profiles were determined in the supernatant with the Bio‐Plex Human Cytokine 17‐Plex Panel, following the manufacturer's instructions (Bio‐Rad). Cytokines in culture medium were also measured as a negative control.

Peripheral blood mononuclear cells (PBMCs) were isolated from buffy coats of healthy blood donors by density gradient centrifugation using Ficoll‐isopaque and frozen in liquid nitrogen until use. Cultured hkPSCs and BM‐MSCs (passages 6–8) of 3 different donors were plated in flat‐bottom 96‐well plates (Costar; Sigma‐Aldrich, St. Louis, MO, https://www.sigmaaldrich.com) and allowed to attach overnight in DMEM‐F12 with 10% normal human serum (NHS). Culture in 10% NHS was chosen because platelet lysates are able to suppress PBMC proliferation on their own (data not shown). PBMCs were stimulated with anti‐CD3/anti‐CD28 Dynabeads (Thermo Fisher) and were seeded in triplicate at a concentration of 1 × 10^5^ cells per well. Stromal cells were added to the PBMC proliferation assay in a ratio of 1:4 and 1:8. ^3^H‐thymidine (0.5 mCi) was added after 5 days and ^3^H‐thymidine incorporation was determined after 16 hours as a measure of proliferation.

### Vascular Plexus Assay

Human umbilical cords were obtained from the Leiden University Medical Center (Leiden, The Netherlands) after informed consent from the parents.

Human umbilical vein endothelial cells (HUVECs) were isolated according to Jaffe et al. 18, with minor modifications: trypsin/EDTA (Sigma‐Aldrich) was used to enzymatically detach the endothelial cells from the vein, and the endothelial cells were cultured on fibronectin‐coated flasks (isolated from bovine plasma; Sigma‐Aldrich) and refreshed twice a week with EC‐medium, consisting of M199 Earl's salt with L‐glutamine (Thermo Fisher), supplemented with 10% (v/v) fetal calf serum (PAA Cell Culture Company, Pasching, Austria, www.paa.com), penicillin/streptomycin (PAA Cell Culture Company), 1,000 IU of heparin (Leo Pharma), and 25 mg bovine pituitary extract (BPE) (Thermo Fisher). HUVECs were used at passages 2–3 18.

Stromal cells and HUVECS were cocultured in a 96‐well plate (Costar; Sigma‐Aldrich) for 1 week in a 4:1 ratio, as has been described previously 19. After 1 week, cells were fixated for 10 minutes with ice‐cold methanol (100%), and endothelial sprouting was visualized with CD31 immunofluorescence (Zeiss LSM500; BD Bioscience). The percentage capillary coverage was analyzed with ImageJ software. NG2 immunofluorescence (BD Bioscience) was determined (Zeiss LSM500) and quantified as mean fluorescent intensity in Image J.

### Transforming Growth Factor‐β Stimulation

hkPSCs and BM‐MSCs were seeded in a density of 200,000 cells in a 6‐well plate and stimulated for 48 hours with 10 ng/ml transforming growth factor (TGF)‐β1 (Preprotech, London, U.K., https://www.peprotech.com). Cells were subsequently trypsinized, permeabilized with 0.1% saponine, and labeled with α‐smooth muscle actin (α‐SMA; BD Bioscience). α‐SMA expression was analyzed with flow cytometry, and mean fluorescent intensities were calculated (Kaluza; Beckman Coulter, Brea, CA, http://ca.beckman.com).

### Kidney Epithelial Wound Scratch Assay

hkPSCs and BM‐MSCs of 3 different donors were plated in a density of 200,000 cells per well in a 6‐well culture plate (Costar; Sigma‐Aldrich) and cultured for 48 hours. HK2 cells were seeded in proximal tubular epithelial cells medium, consisting of a 1:1 ratio of DMEM and Ham's F‐12 (Lonza), supplemented with insulin (5 µg/ml), transferrin (5 µg/ml), selenium (5 ng/ml), hydrocortisone (36 ng/ml), triiodothyrinine (40 pg/ml), and epidermal growth factor (10 ng/ml) (Sigma‐Aldrich) in a density of 500,000 cells per well in a 6‐well cell culture plate (Costar) and cultured until confluent. A scratch wound was created in the monolayer of HK2 cells using a 200‐µl pipette tip. After the scratch, cells were washed with phosphate‐buffered solution (PBS) and provided either with fresh medium (αMEM 5% PL) or with complete conditioned medium from either hkPSCs or BM‐MSCs. Scratches were imaged at 4, 7, 14, and 28 hours at the same position in duplicates with an inverted bright‐field microscope (Leica DFC 295; Leica Biosystems). The scratch area was measured at each time point using ImageJ software, and the percentage wound closure was calculated.

Growth factors in the conditioned medium of kPSCs were measured using a custom‐made growth‐factor panel, following the manufacturer's instructions (R&D Systems, Minneapolis, MN, https://www.rndsystems.com). The hepatocyte growth factor (HGF)‐receptor was blocked with an HGF receptor/c‐MET antibody (R&D Systems) in a concentration of 1 µg/ml 1 hour before the wound scratch assay and after adding the hkPSC supernatant. In the control conditions an isotype antibody (goat IgG, homemade) was added.

### Neonatal Injection Model

Animal experiments were approved by the University of Queensland animal ethics committee and adhered to the Australian Code of Practice for the Care and Use of Animals for Scientific Purposes. Neonates of outbred CD1 mice were used for neonatal injection. hkPSCs and BM‐MSCs of 3 and 2 different donors, respectively, were injected into the neonatal kidneys at postnatal day 1 by using a microinjection pipet in a protocol adapted from the protocol previously described 16. In short, neonates were anesthetized and a small incision in the skin was made. Cells were resuspended in PBS and mixed with Fluoresbrite Yellow Green microspheres (2.0 µm; Polyscience, Niles, IL, https://www.polyscience.com) in a ratio of 1:50 for identification of injection sites in the neonatal kidney. Using an Eppendorf microinjector, we injected cells into the kidney through the muscle layer in a volume of 300 nl, corresponding with 3,000–5,000 cells. Kidneys were harvested at 4 days postinjection. In total, seven mice were analyzed: three mice with hkPSCs from three different donors; three mice with BM‐MSCs from two different donors, and one sham‐operated mouse. In all mice, injection into the kidney was confirmed by fluorescence of the coinjected microspheres.

### Glycerol‐Induced Rhabdomyolysis Model of Acute Kidney Injury

Animal experiments were performed in accordance with institutional, regional, and state guidelines and in adherence to the Italian National Institutes of Health Guide for the Care and Use of Laboratory Animals. Rhabdomyolysis‐induced acute kidney injury was studied in 6‐week‐old male C57Bl/6 mice (Envigo RMS Srl, San Pietro al Natisone, Italy, http://www.envigo.com) by intramuscular injection on day 0 with hypertonic glycerol (8 ml/kg body weight of a 50% glycerol solution; Sigma‐Aldrich) into the inferior hind limbs, as has been described previously 20. hkPSCs were labeled with the PKH26 Red Fluorescence Cell Linker Kit (Sigma‐Aldrich), according to the manufacturer's instructions, and labeling efficiency was verified by flow cytometry. Cells were acquired before and after labeling with a MACSQuant Analyzer Flow Cytometer and analyzed with MACSQuantify software (Miltenyi Biotec GmbH). Cell vitality was monitored using propidium iodide staining, and we observed >95% living cells. The mice received PKH26‐labeled hkPSC following one of two injection routes. For subcapsular injection, 4 hours following kidney injury, mice (*n* = 6 for blood urea nitrogen [BUN] measurement, *n* = 4 for confocal microscopy) were anesthetized with Avertin (2,2,2‐tribromoethanol, 250 mg/kg; Sigma‐Aldrich) and subjected to dorsal incision on the left side to exteriorize the left kidney. A 1‐mm incision was made in the capsule of the kidney, and 750,000 cells were injected into 25‐µl of sterile PBS with a Hamilton syringe equipped with a 27‐G blunt‐ended needle. After cell infusion, the kidney capsule was cauterized with an electric scalpel, and the dorsal incision was sutured. The mouse was rehydrated with subcutaneous injection of 500 μl saline solution and maintained in a warm environment for 2 hours postsurgery. Control mice were injected with saline solution (*n* = 6 for BUN measurement, *n* = 4 for confocal microscopy). For intravenous retro‐orbital injection, 4 hours and 24 hours following kidney injury, mice (*n* = 6 for BUN measurement, *n* = 4 for confocal microscopy) were anesthetized with isoflurane (Aerrane; Baxter, Rome, Italy, http://www.baxteritalia.it) and injected retro‐orbitally through the venous plexus with 750,000 cells in 150 µl of sterile PBS each time using a 27‐G needle. Control mice were injected with saline solution (*n* = 8 for BUN measurement, *n* = 4 for confocal microscopy).

Blood samples were obtained from the submandibular venous sinus at days 0, 4, 6, and 14, and BUN levels were measured by Reflotron System (Roche Diagnostics, Rotkreuz, Switzerland, www.roche.com). Four animals per group were sacrificed at day 6, and kidney, lungs, and liver were harvested for confocal microscopy.

### Immunofluorescence of Kidney Sections

In the neonatal injection model, kidney samples were fixed in 4% PFA, followed by 30% sucrose overnight and embedded in TissueTek OCT compound (Sakura Finetek, Torrance, CA, http://www.sakura‐americas.com). Samples were frozen in liquid nitrogen and stored at −80°C. Ten‐micrometer‐thick sections were cut and postfixed with 4% PFA for 10 minutes at room temperature. Stainings were performed using the manufacturer's protocol (Mouse on Mouse kit; Vector Laboratories, Burlingame, CA, https://vectorlabs.com; Brunschwig Chemie, Amsterdam, The Netherlands, http://www.brunschwig.nl). Samples were stained with antibodies against human mitochondria, nuclei, and collagen IV (Abcam, Cambridge, U.K., http://www.abcam.com) and analyzed using a TCS SP8 laser confocal microscope (Leica Biosystems). In the rhabdomyolysis‐induced acute kidney injury model, confocal microscopy was performed on 10‐ μm sections of renal frozen tissues using a TCS SP5‐II laser confocal microscope (Leica Biosystems). Staining for fluorescein isothiocyanate (FITC)‐labeled Dolichos biflorus agglutinin and FITC‐labeled Lotus tetragonolobus agglutinin (Vector Laboratories) was performed following manufacturer's instructions. To‐pro‐3 (Thermo Fisher) was used for counterstaining nuclei.

### Statistical Analysis

Differences between two groups were analyzed using an unpaired two‐sample Student *t* test. When more than two groups were analyzed, a two‐way analysis of variance test was used with Bonferroni's comparison test as a post hoc test. Differences were considered statistically significant when *p* < .05. Data analysis was performed using GraphPad Prism, version 5.0 (Graphpad Prism Software, Inc., La Jolla, CA, https://www.graphpad.com). For statistical analysis of the microarray data, *p* values were corrected for multiple testing according to Benjamini and Hochberg 17.

## Results

### A Novel Method to Isolate Clinical‐Grade Human Kidney‐Derived Perivascular Stromal Cells

**Figure 1:**
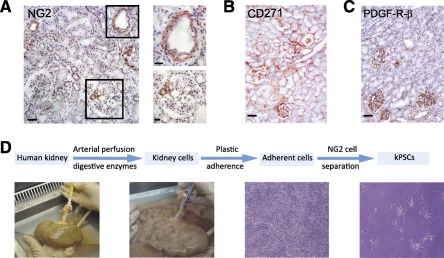
Isolation method of human kPSCs. **(A):** NG2 is expressed in the human kidney mainly around the arteries, arterioles and afferent and efferent arterioles of the glomerulus. CD271 **(B)** and PDGF‐R‐β **(C)** are also expressed within the glomeruli and around the peritubular capillaries. **(D):** Schematic representation of the isolation method. In order to obtain NG2‐positive hkPSCs, human kidneys are continuously perfused with digestive enzymes to single kidney cells. Afterward, crude kidney cell suspensions were cultured on plastic for selection on plastic adherence and subsequently sorted for NG2 positivity, resulting in the appearance of spindle‐ shaped cells. Scale bars = 50 μm (**[A]**, left), 20 μm (**[A]**, right), and 50 μm **(B, C)**. Abbreviations: kPSCs, kidney‐derived perivascular stromal cells; PDGF‐R‐β, platelet‐derived growth factor‐receptor‐β.

In order to evaluate whether hkPSCs are a potential new cell source for use in cell therapy to treat kidney disease in a clinical setting, we chose to develop a clinical‐grade acceptable SOP with the use of clinical‐grade materials and enzymes. This protocol is developed on the basis of the pancreatic islet isolation protocol currently in use for clinical application in our center 21. Perivascular stromal cells were isolated on the basis of NG2 expression. NG2 is an integral membrane proteoglycan that is associated with perivascular cells during vascular morphogenesis 22. Within the human kidney, NG2 is mainly expressed around the large arteries and the afferent and efferent arterioles (Fig. 1A). NG2 expression is more restricted than is the expression of CD271, an enrichment marker for BM‐MSCs (Fig. 1B) 23 or of PDGF‐R‐β‐positive perivascular cells (Fig. 1C). These markers are also expressed within the glomeruli and around the peritubular capillaries of the kidney. NG2‐positive cells were isolated from a pool of 10 transplant‐grade kidneys discarded for surgical reasons. Experiments were performed, and results are shown for three different donors. The average donor age was 62 years, with an average estimated creatinine clearance (Cockroft) of 105 ml per minute. In order to isolate NG2‐positive cells, we perfused these kidneys with collagenase to dissociate into single cells, enriched on the basis of plastic adherence, then sorted on the basis of NG2 expression at the first passage (Fig. 1D).

### Characterization of Human Kidney‐Derived Perivascular Stromal Cells

**Figure 2:**
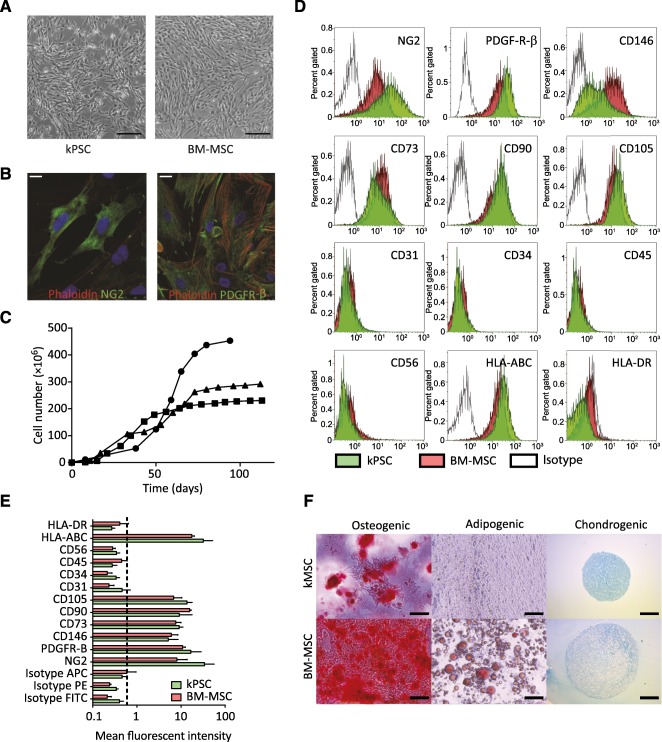
Characterization of human kPSCs. **(A):** Human kPSCs have a morphology similar to that of BM‐MSCs and are positive for NG2 and PDGF‐R‐β, as is shown with confocal images **(B)**. **(C):** Growth characteristics of human kPSCs. **(D):** Representative FACS analysis shows that human kPSCs are positive for the pericytic markers NG2, PDGF‐R‐β, and CD146 and for the MSC markers CD73, CD90, and CD105, and are negative for CD31, CD34, CD45, and CD56. Human kPSCs express type I human leukocyte antigen (HLA‐ABC) and are negative for type II HLA (HLA‐DR). **(E):** Mean fluorescent intensity of the different markers (*n* = 3 donors). **(F):** Trilineage differentiation. Human kPSCs differentiate into bone and cartilage but not into adipocytes. Scale bars = 200 µm **(A, F)** and 20 µm **(B)**. Abbreviations: BM‐MSC, bone marrow‐derived mesenchymal stromal cells; FITC, fluorescein isothiocyanate; HLA, human leukocyte antigen; kMSC, kidney‐derived mesenchymal stromal cells; kPSC, kidney‐derived perivascular stromal cells; PDGF‐R‐β, platelet‐derived growth factor‐receptor‐β.

NG2‐positive hkPSCs showed a bright‐field morphology similar to that of BM‐MSCs (Fig. 2A) and, as with BM‐MSCs, were positive for the pericyte markers NG2 and PDGF‐R‐β, as is shown with confocal microscopy (Fig. 2B). Growth characteristics are shown from flow cytometry‐confirmed NG2 homogeneous populations; at around passage 9, hkPSCs reached senescence (Fig. 2C). In addition to NG2, hkPSCs were positive for the surface markers PDGF‐R‐β, CD146, CD73, CD90, and CD105 while being negative for CD31, CD34, CD45, and CD56, as determined by FACS (Fig. 2D). This marker expression is robust, as depicted by the mean fluorescent intensity of cells of three different donors (Fig. 2E). Whereas human BM‐MSCs were able to differentiate into all three lineages, hkPSCs could differentiate toward osteocytes and chondrocytes, and no adipogenic differentiation was observed (biological triplicates) (Fig. 2F).

### Organ‐Specific Gene Expression Profile of Human Kidney‐Derived Perivascular Stromal Cells

**Figure 3:**
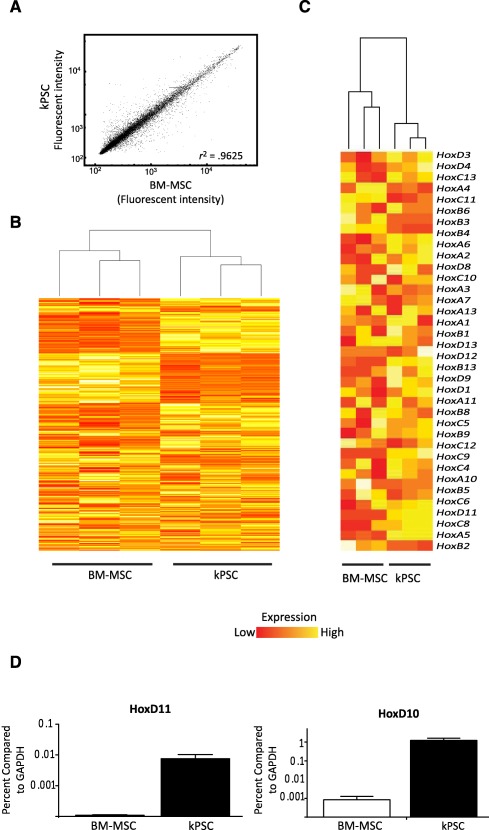
Transcriptome analysis of human kPSCs in comparison with BM‐MSCs. **(A):** When comparing all transcripts (35,000), BM‐MSCs and human kPSCs show a similar expression profile, as is depicted by a Pearson correlation score of 0.9625. **(B):** Human kPSCs and BM‐MSCs show a hierarchical clustering per cell type. **(C):** Hierarchical clustering is also observed in Hox genes. **(D):** Differential expression of HoxD11 and HoxD10, homeobox factors important for nephrogenesis, as confirmed by polymerase chain reaction. (GEO accession GSE77227, http://www.ncbi.nlm.nih.gov/geo/query/acc.cgi?acc=GSE77227.) Abbreviations: BM‐MSC, bone marrow‐derived mesenchymal stromal cells; GAPDH, glyceraldehyde‐3‐phosphate dehydrogenase; kPSC, kidney‐derived perivascular stromal cells.

In order to further compare hkPSCs to BM‐MSCs, we performed Illumina microarray expression profiling on biological triplicates of different donors. Analysis of expression levels of 35,000 transcripts showed that most genes showed a similar expression level (Pearson correlation coefficient of 0.9625), suggesting high similarity between the two cell types (Fig. 3A). However, 2,600 genes were differentially expressed, and hierarchical clustering was able to distinguish on the basis of cell source (Fig. 3B). Table 1 shows the top five up‐ and downregulated genes comparing BM‐MSCs and hkPSCs on the basis of differential *p* value. The top 50 up‐ and downregulated genes can be found in supplemental online Table 1. Interestingly, homeobox factor HoxD11 is in the top five most upregulated genes in hkPSCs. Homeobox transcription factors, which are important in anatomical patterning during development, showed hierarchical clustering when comparing BM‐MSCs with hkPSCs (Fig. 3C). Homeobox paralogues Hox10 and Hox11 are important in kidney development 24, 25. Both genes are highly expressed in hkPSCs but not in BM‐MSCs, as confirmed by PCR (Fig. 3D). These results indicate that although hkPSCs and BM‐MSCs may display a similar phenotype of surface markers, there are tissue‐specific differences in expression profile between the cell types.

**Table 1:** Top five differentially expressed genes comparing BM‐MSCs to hkPSCs

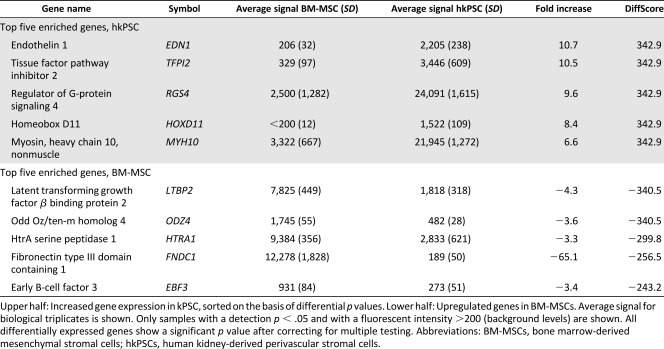

### Immunomodulatory Capacity of Human Kidney‐Derived Perivascular Stromal Cells

**Figure 4:**
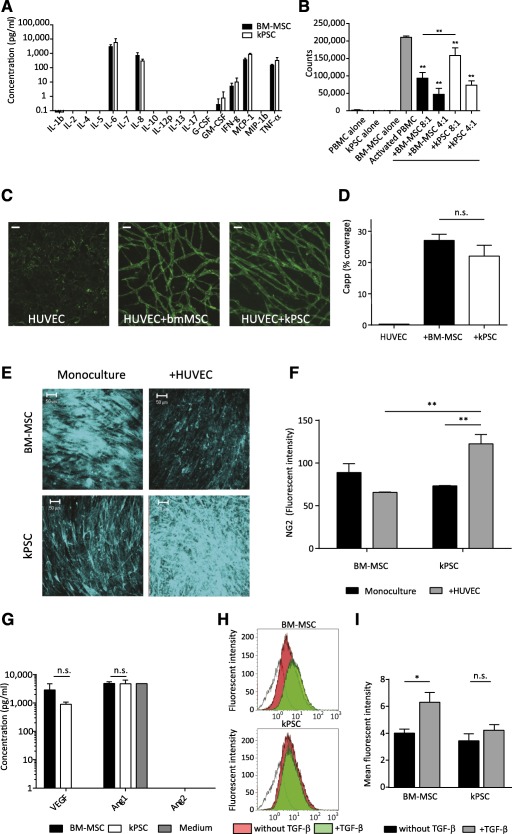
Immunomodulatory and vascular stabilization function of human kPSCs. **(A):** Cytokine expression profile of unstimulated human kPSCs and BM‐MSCs. **(B):** Peripheral blood mononuclear cells (PBMCs) suppression assay. Proliferation of activated PBMCs was decreased when cocultured with both hkPSCs and BM‐MSCs in a dose‐dependent matter. (Ratio is number of activated PBMCs vs. number of MSCs.) **(C):** When human umbilical vein endothelial cells are cocultured with either human kPSCs or BM‐MSCs, endothelial networks were formed, which was not observed in monoculture. There were no significant differences in vascular plexus formation comparing coculture with BM‐MSCs or human kPSCs **(D)**. **(E):** When human kPSCs were cocultured with endothelial cells, the expression of NG2 was increased; this was not seen with BM‐MSCs. **(F):** Quantification of NG2 positivity with and without coculture with endothelial cells. **(G):** No differences were observed in excretion of vascular growth factors by human kPSCs in comparison with BM‐MSCs. **(H):** After stimulation of human kPSCs with 10 ng/ml TGF‐β, there was no increase in α‐smooth muscle actin (SMA) expression. This was not observed for BM‐MSCs. Isotype control (white), unstimulated cells (red), cells stimulated with 10 ng/ml TGF‐β (green). **(I):** Quantification of α‐SMA positivity; *n* = 3 biological triplicates. Scale bar = 50 µm **(C, E)**. ∗, *p* < .05; ∗∗, *p* < .001. Abbreviations: Ang, angiopoietin; bmMSC, BM‐MSC, bone marrow‐derived mesenchymal stromal cells; capp, capillary coverage; G‐CSF, granulocyte colony‐stimulating factor; GM‐CSF, granulocyte‐macrophage colony‐stimulating factor; HUVEC, human umbilical vein endothelial cells; IFN, interferon; IL, interleukin; kPSC, kidney‐derived perivascular stromal cells; MIP‐1, macrophage inflammatory protein 1; MCP‐1, monocyte chemoattractant protein 1; n.s., nonsignificant; PBMC, peripheral blood mononuclear cells; TGF, transforming growth factor; TNF‐α, tumor necrosis factor‐α; VEGF, vascular endothelial growth factor.

One important and extensively studied feature of MSCs is their anti‐inflammatory and immunomodulatory potential. MSCs are able to regulate proliferation and cytotoxicity of T cells, macrophages, and B cells, which are also major players in kidney disease and transplantation, as has been reviewed elsewhere 26. Therefore, we evaluated the immunomodulatory potential of hkPSCs in comparison with BM‐MSCs. Unstimulated human kPSCs and BM‐MSCs showed a similar expression profile for all major cytokines (Fig. 4A). We also evaluated the immunosuppressive capacity of hkPSCs. In a peripheral blood mononuclear cell (PBMC) suppression assay, where PBMCs were activated by polyclonal CD3/CD28 activation in the absence or presence of stromal cells, both hkPSCs and BM‐MSCs inhibited proliferation in a dose‐dependent manner (Fig. 4B). However, BM‐MSCs were more potent in inhibiting proliferation at a lower cell ratio (8:1 PBMCs:MSC ratio) in comparison with hkPSCs.

### Coculture of Endothelial Cells With Human Kidney‐Derived Perivascular Stromal Cells Stabilizes Vascular Network Formation

To evaluate whether cultured hkPSCs still have pericytic properties, we cocultured cells with human umbilical vein endothelial cells (HUVEC). HUVECs were not able to form endothelial sprouts in monoculture. However, when cocultured with either BM‐MSCs or hkPSCs, vascular plexus formation occurred (Fig. 4C). There were no significant differences in vascular network formation between the cell types (Fig. 4D). Interestingly, when cocultured with endothelial cells, hkPSCs showed an increase in NG2 expression, although this was not observed with BM‐MSCs (Fig. 4E, 4F). Both BM‐MSCs and hkPSCs mainly secreted vascular endothelial growth factor (VEGF), and there was no difference between the cell types in the levels of VEGF, angiopoetin 1, and angiopoetin 2 produced (Fig. 4G).

### hkPSCs Do not Become Myofibroblasts After Stimulation With TGF‐β

Because stromal cells in general have the capacity to become myofibroblasts after stimulation with TGF‐β, thereby contributing to fibrosis, we evaluated whether hkPSCs have this capacity. Interestingly, stimulation of hkPSCs with TGF‐β did not increase α‐SMA expression, suggesting that hkPSCs did not become myofibroblasts (Fig. 4H, 4I).

### Enhanced Renal Epithelial Wound Repair Capacity of Human Kidney‐Derived Perivascular Stromal Cells

**Figure 5:**
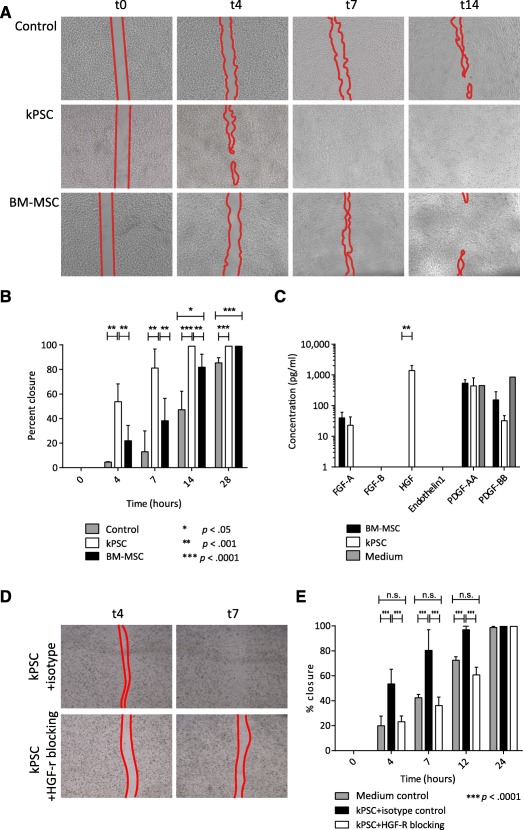
Human kPSCs are able to enhance epithelial repair in a wound scratch assay. **(A):** Bright‐field images showing representative images of the wound scratch assay in control medium or conditioned medium of human kPSCs and BM‐MSCs across a time period of 14 hours. **(B):** Quantification of the rate of wound closure in all three conditions after 0, 4, 7, 14, and 28 hours. The wound closes significantly faster in the presence of human kPSC‐conditioned medium in comparison with BM‐MSC‐conditioned medium or control medium. **(C):** Excretion of growth factors in the conditioned medium. HGF is excreted by human kPSCs but not by BM‐MSCs. **(D):** Bright‐field images showing representative images of the wound scratch assay with and without HGF‐R blocking. **(E):** Quantification of the rate of wound closure after 0, 4, 7, 12, and 24 hours; *n* = 3 biological triplicates. ∗, *p* < .05; ∗∗, *p* < .001; ∗∗∗, *p* < .0001. Abbreviations: BM‐MSC, bone marrow‐derived mesenchymal stromal cell; FGF, fibroblast growth factor; HGF, hepatocyte growth factor; kPSC, kidney‐derived perivascular stromal cell; n.s., nonsignificant; PDGF, platelet‐derived growth factor; t, time.

In order to evaluate the effect of hkPSCs on renal tubular epithelial repair, we made a scratch in a monolayer of human kidney proximal tubular epithelial cells (HK2). Under control culture conditions, at least 28 hours was necessary for 80% wound closure. Interestingly, when conditioned media from hkPSCs were added, significant closure was already observed after 4 hours, with 80% of the wound closed after 7 hours (mean of duplicate experiments from three different donors). Importantly, 14 hours was required to reach an 80% wound closure in parallel experiments with the conditioned media from BM‐MSCs (Fig. 5A, 5B). This shows that hkPSCs can produce factors able to better support renal epithelial repair. HGF is most likely an important factor in this activity because HGF was secreted at high levels by hkPSCs but not by BM‐MSCs, whereas no differences were observed in the levels of other growth factors (PDGF‐AA, PDGF‐BB, endothelin1, fibroblast growth factor [FGF]‐a, FGF‐B) (Fig. 5C). Moreover, blocking the HGF receptor on the epithelial cells resulted in decreased wound healing (Fig. 5D, 5E).

### Interstitial Integration and Survival of Human Kidney‐Derived Perivascular Stromal Cells in the Neonatal Kidney

**Figure 6:**
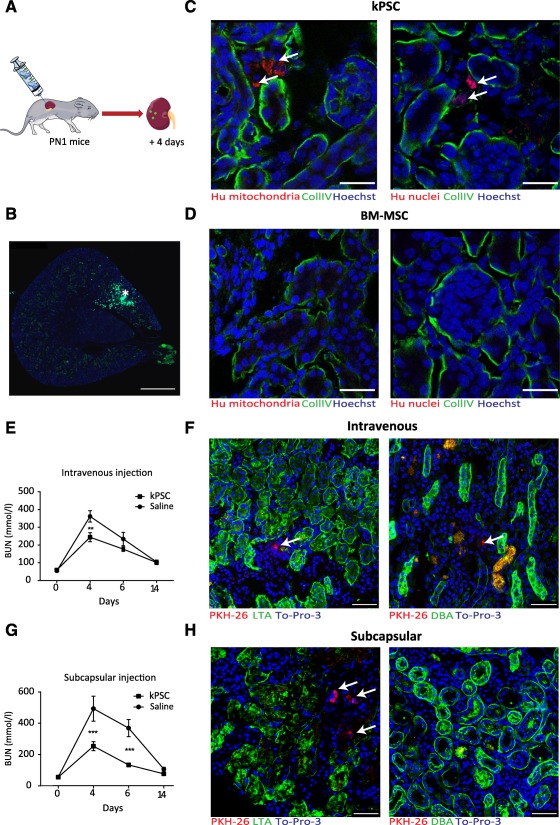
Human kPSCs are able to survive and integrate into the kidney interstitium in a neonatal injection method and in a model of acute kidney injury and give preservation of renal function. **(A):** Cells and fluorescent microspheres were injected into the kidney of neonatal mice (postnatal day 1, PND1). **(B):** Injection into the kidney was confirmed by fluorescent microspheric beads (∗). **(C):** Human kPSCs injected into the kidneys of neonatal mice (PND1) were localized in the cortical interstitium, as is shown with specific antibodies for human mitochondria and human nuclei, respectively (arrows). **(D):** No BM‐MSC could be found in the kidney 4 days after injection. **(E):** BUN measurements after i.v. injection of human kPSCs in a glycerol‐induced rhabdomyolysis model. **(F):** Human kPSCs were localized mainly in the interstium of the medulla after i.v. infusion and did not integrate in the Lotus tetragonolobus agglutinin‐positive proximal tubuli or Dolichos biflorus agglutinin‐positive collection duct (arrows). **(G):** BUN after subcapsular administration of human kPSCs in a glycerol‐induced rhabdomyolysis model. **(H):** Human kPSCs integrated in the interstium in the cortex and not in the medulla (arrows). No integration in the tubuli was observed. Scale bars = 500 μm **(B)** and 50 μm **(C, D, F, H)**. Abbreviations: BM‐MSC, bone marrow‐derived mesenchymal stromal cell; BUN, blood urea nitrogen; kPSC, kidney‐derived perivascular stromal cell.

To determine the role of hkPSCs in kidney development, we injected hkPSCs into neonatal mice at postnatal day 1 using microinjection (Fig. 6A, 6B). Interestingly, at day 4 postinjection, human kPSCS were able to integrate and survive within the cortical, but not the medullary interstitium of the mouse kidney with no evidence of rejection (Fig. 6C). No such persistence was observed when human BM‐MSCs were injected (Fig. 6D), consistent with our previous studies 16. No integration into tubular structures was seen with either human BM‐MSCs or hkPSCs upon injection. The persistence of viable human hkPSCs for 4 days within the renal interstitium suggests a differential integration capacity for this stromal cell type.

### Human Kidney‐Derived Perivascular Stromal Cells Integrate in the Renal Interstitium and Improve Renal Function in a Glycerol‐Induced Rhabdomyolysis Acute Kidney Injury Model

To access the effect of hkPSCs in kidney injury, we injected the cells in a glycerol‐induced rhabdomyolysis acute kidney injury model. We evaluated the effect of hkPSCs via two routes of administration: renal subcapsular and intravenous. Blood ureum nitrogen (BUN) measurements show that at the peak of acute kidney injury (AKI), renal function was significantly preserved in the hkPSC‐treated group, independent of route of delivery (Fig. 6E, 6G). After both routes of delivery, interstitial integration of the PKH26‐labeled hkPSCs was observed within the kidney, although fewer cells were observed after intravenous injection (0.0247 ± 0.002 cells per field subcapsular vs. 0.0164 ± 0.0051 cells per field i.v.). After subcapsular injection, cells were mainly distributed in an area of 990 µm around the area of injection. After intravenous injections, most cells were observed in the medulla region and fewer in the cortex (0.0203 ± 0.006 vs. 0.0095 ± 0.0066) (Fig. 6F, 6H).

## Discussion

It has previously been reported that perivascular stromal cells from several different human organs share features with MSCs 10. More recently, it has been shown that organ‐derived perivascular cells may exhibit tissue‐specific functions 27. Human myocardial perivascular cells, for example, stimulate angiogenic responses under hypoxia and differentiate into cardiomyocytes in vivo, characteristics not seen in perivascular cells isolated from other tissues 28.

Here we sought to isolate human kidney‐derived perivascular stromal cells and show that these cells have distinct properties in comparison with human BM‐MSCs. In order to evaluate the functionality of these cells, we studied their interaction with several different renal cell types, including endothelial cells, proximal tubular cells, and (infiltrating) immune cells. There were similarities between human BM‐MSCs and hkPSCs; however, hkPSCs showed a distinct mRNA expression profile, did not differentiate into adipocytes, and displayed a more potent kidney epithelial wound‐healing capacity. Furthermore, hkPSCs were able to integrate into the interstitium of the developing kidney, whereas BM‐MSCs did not. Finally, hkPSCs could preserve kidney function in a glycerol‐induced rhabdomyolysis model of acute kidney injury.

Because there is a lack of specific markers of MSCs, the International Society for Cellular Therapy has proposed criteria to define MSCs. These include plastic adherence; the marker expression of CD73, CD90, and CD105 while being negative for CD14, CD34, and CD45; and the capacity to differentiate into bone, cartilage, and fat in vitro 1. The hkPSCs we isolated did not fulfill these criteria because there was no adipocyte differentiation. However, these criteria are based on bone marrow‐derived MSCs, and the lack of this capacity might actually be beneficial because it has previously been shown that MSCs injected into the renal artery in a glomerulonephritis model can turn into adipocytes in the glomeruli accompanied by glomerular sclerosis around these adipocytes 29.

MSCs are important for tissue homeostasis, most likely via the secretion of several soluble factors and microvesicles containing, among others, mRNAs and miRNAs 30, 31. Indeed, BM‐MSC‐conditioned medium is able to accelerate wound healing in vitro 32, 33 and, in a mouse model of glycerol‐induced acute kidney injury, enhanced recovery in kidney function was observed when either MSCs or MSC‐derived microvesicles were injected 30. The same is most likely true for organ‐derived perivascular stromal cells. Conditioned medium from murine kidney MSC‐like cells was able to enhance kidney epithelial wound healing in an in vitro wound scratch assay 16. Here we show that the conditioned medium from human kPSCs elicited accelerated repair in a tubular epithelial wound scratch assay, suggesting tissue‐specific paracrine signaling. An important factor in this signaling is most likely hepatic growth factor (HGF), because HGF is highly secreted by kPSCs and not by BM‐MSCs. Moreover, after blocking of the HGF‐receptor, the effect of hkPSCs on wound healing was diminished. HGF has long been recognized as an important factor in kidney regeneration and attenuation of renal fibrosis in several different animal models 34. HGF is primarily produced in nonepithelial cells, such as fibroblasts and pericytes, and is able to block myofibroblast activation and therefore renal fibrosis. Moreover, HGF can prevent tubular epithelial cell death both in vitro and in vivo, the latter resulting in improved renal function after acute kidney injury 35, 36, 37. HGF is most likely also important in human kidney regeneration, because a high expression of HGF in protocol biopsies after kidney transplantation correlated with lower levels of fibrosis 38.

Although this study is not the first to describe an MSC‐like population in the human kidney 39, hkPSCs isolated through the current protocol represent a distinct population. Resident kidney MSCs, as described by Bruno et al. 39, were isolated from the glomeruli, whereas the hkPSCs described in the current protocol were isolated on the basis of NG2 expression. Because NG2 is mainly expressed around the large vessels (Fig. 2A), this is more likely to represent a different perivascular population. Another major difference is that hkPSCs are not positive for CD24, Pax2, Oct4, or NANOG (supplemental online Fig. 1) and are, in contrast to glomerular MSCs, not able to differentiate into adipocytes.

The differences between human kPSCs and BM‐MSCs mentioned above may reflect differences in imprinting by the tissue of origin. This was previously also observed for fibroblasts isolated from different organs 40. In the current study, the expression profile of homeobox genes, which are important for anatomical patterning, showed a differential upregulation of HoxD10 and HoxD11 expression by hkPSC, with the latter gene in the top five most differentially expressed genes. Both Hox10 and Hox11 are crucial for nephrogenesis. Hox10 genes function in the differentiation and integration of the FoxD1^+^ renal cortical stroma, and Hox11 genes are expressed in the metanephric mesenchyme. Loss of either Hox10 or Hox11 gene function results in the loss of ureteric bud induction, reduced branching, and decreased nephrogenesis, phenotypes only described for Hox10 and 11 mutants and not for other Hox mutants 24, 25. This potential tissue “memory of origin” may be reflected in the observed differences in potential to integrate back into the kidney when injected into the renal parenchyma. Such integration was never observed for the bone marrow MSCs, while the hkPSCs migrated into the renal interstitium and survived. Although we observed integration into the cortical interstitial compartment, we did not observe any integration into the epithelium. Previously, it was reported that murine kidney‐derived MSC‐like cells can integrate into the developing collecting duct. However, the human kPSCs were isolated from the perivascular fraction on the basis of NG2 expression, while Li et al. isolated murine MSC‐like cells on the basis of HoxB7 expression and thus the collecting duct epithelial compartment 16. When looking at the expression pattern of the homeobox factors, HoxB7 expression in the human kPSCs was low, and HoxD11 expression was high, suggesting a different origin likely reflected in their distinct integration capacity and function. It remains possible that a human kidney MSC‐like population similar to that isolated from mouse or reciprocally a murine kPSC population similar to the hkPSC described here may exist.

Interestingly, hkPSCs were able to integrate back into the renal interstitium and reduce the severity of kidney injury in vivo in a rhabdomyolysis model. This was independent of the route of delivery because both subcapsular and intravenously injected hkPSCs were able to preserve renal function. However, with the subcapsular injections, integration of the hkPSCs into the interstitium appeared more pronounced, as did the renoprotective effect.

Whether human kPSCs are more suitable for cell therapy for kidney diseases than BM‐MSCs, which are currently studied in clinical trials, still remains to be further elucidated. Their capacity to reintegrate into the renal stroma, to improve tubular epithelial wound repair, and to preserve function in an AKI model would suggest that hkPSCs may have an organotypic role in maintaining and restoring renal interstitial homeostasis without showing the risk of increasing fibrosis. To be able to compare these cell types for future cell therapy purposes, we chose to isolate the hkPSCs with a SOP in a clinical‐grade manner, with clinical‐grade enzymes, materials, and methods that would allow direct translation into a clinical product. Using this protocol, one can obtain large quantities of clinical‐grade isolated hkPSCs. The yield from one human kidney would be an average of 2.7 × 10^12^ hkPSCs after passage 6. Hence, one donor kidney could yield a sufficient number of hkPSCs for the treatment of several different patients as a cell therapy. This makes human kPSCs an interesting new cell source to develop for regenerative medicine for kidney diseases.

## Conclusion

These data show that human kPSCs show distinct functional properties in comparison with BM‐MSCs with respect to growth factor excretion, interstitial integration, and amelioration of kidney injury, suggesting significant potential for hkPSCs and their secretome as therapeutic candidates for the treatment of kidney disease.

## Author Contributions

D.G.L.: conception and design, collection and/or assembly of data, data analysis and interpretation, manuscript writing, final approval of manuscript; M.E.J.R., P.R., C.v.K., and M.H.L.: conception and design, data analysis and interpretation, manuscript writing; J.L., A.J.P., and M.A.E.: conception and design, collection and/or assembly of data, data analysis and interpretation, manuscript writing; E.L.: collection and/or assembly of data, data analysis and interpretation; H.C.d.B.: collection and/or assembly of data, data analysis and interpretation, manuscript writing; W.E.F.: conception and design, manuscript writing; T.J.R.: conception and design, data analysis and interpretation, manuscript writing, financial support.

## Disclosure of Potential Conflicts of Interest

M.H.L. is a patent holder and has received honoraria as an invited speaker for the Amgen Symposium, Melbourne, Australia, 2016. D.G.L., M.A.E., and T.J.R. are on a patent application regarding this subject. The other authors indicated no potential conflicts of interest.

## Supporting information

Supporting Information
